# Supplementary material for: Reversal of Biological Age in Multiple Rat Organs by Young Porcine Plasma Fraction
Source: bioRxiv. 2023 Aug 7:2023.08.06.552148. Preprint. [Version 1] doi: 10.1101/2023.08.06.552148 (PMC10441355; doi:10.1101/2023.08.06.552148)
Supplement: Supplement 2 [file NIHPP2023.08.06.552148v1-supplement-2.pdf]

bioRxiv preprint doi: https://doi.org/10.1101/2023.08.06.552148; this version posted August 7, 2023. The copyright holder for this preprint (which was not certified by peer review) is the author/funder, who has granted bioRxiv a license to display the preprint in perpetuity. It is made available under aCC-BY 4.0 International license.

Supplementary Material
for the article "***Reversal of Biological Age in Multiple Rat Organs by Young Plasma Fraction***"
by S. Horvath et al. Harold Katcher

The Supplement contains Supplementary Figures, Supplementary Tables, Supplementary Methods and software code for the rat clocks.

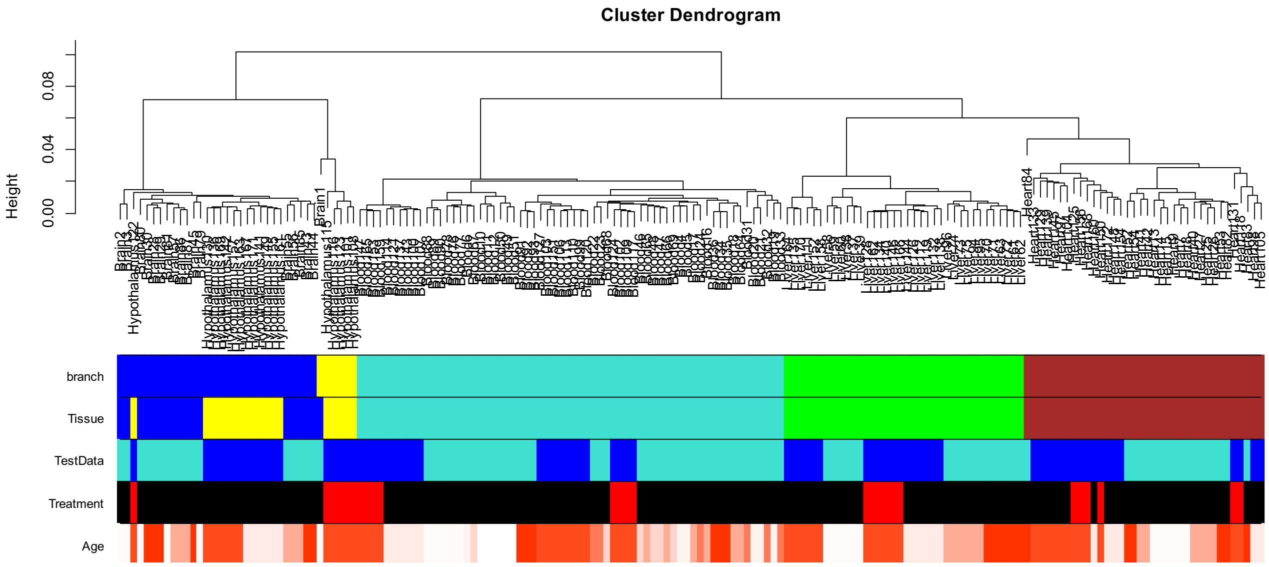

Supplementary Figure 1. Unsupervised Hierarchical Clustering of Rat Tissue Samples Based on Inter-array Correlations. This figure visualizes the unsupervised hierarchical clustering of rat tissue samples, generated using average linkage clustering based on 1 minus the Pearson correlation as dissimilarity measure (utilizing the R hClust function). Five color-coded bands below the dendrogram provide additional information: The first band denotes the cluster branch, indicating the grouping of samples. The second band specifies tissue types, represented as blue for brain, yellow for hypothalamus, turquoise for blood, green for liver, and brown for heart. The third band identifies the test data utilized to evaluate the E5 treatment, marked in blue. The fourth band signifies samples that underwent the E5 treatment, represented in red. The fifth band differentiates age groups: white symbolizes young samples, while red stands for older ones. Notably, the branching within the dendrogram closely aligns with tissue types.

bioRxiv preprint doi: https://doi.org/10.1101/2023.08.06.552148; this version posted August 7, 2023. The copyright holder for this preprint (which was not certified by peer review) is the author/funder, who has granted bioRxiv a license to display the preprint in perpetuity. It is made available under aCC-BY 4.0 International license.

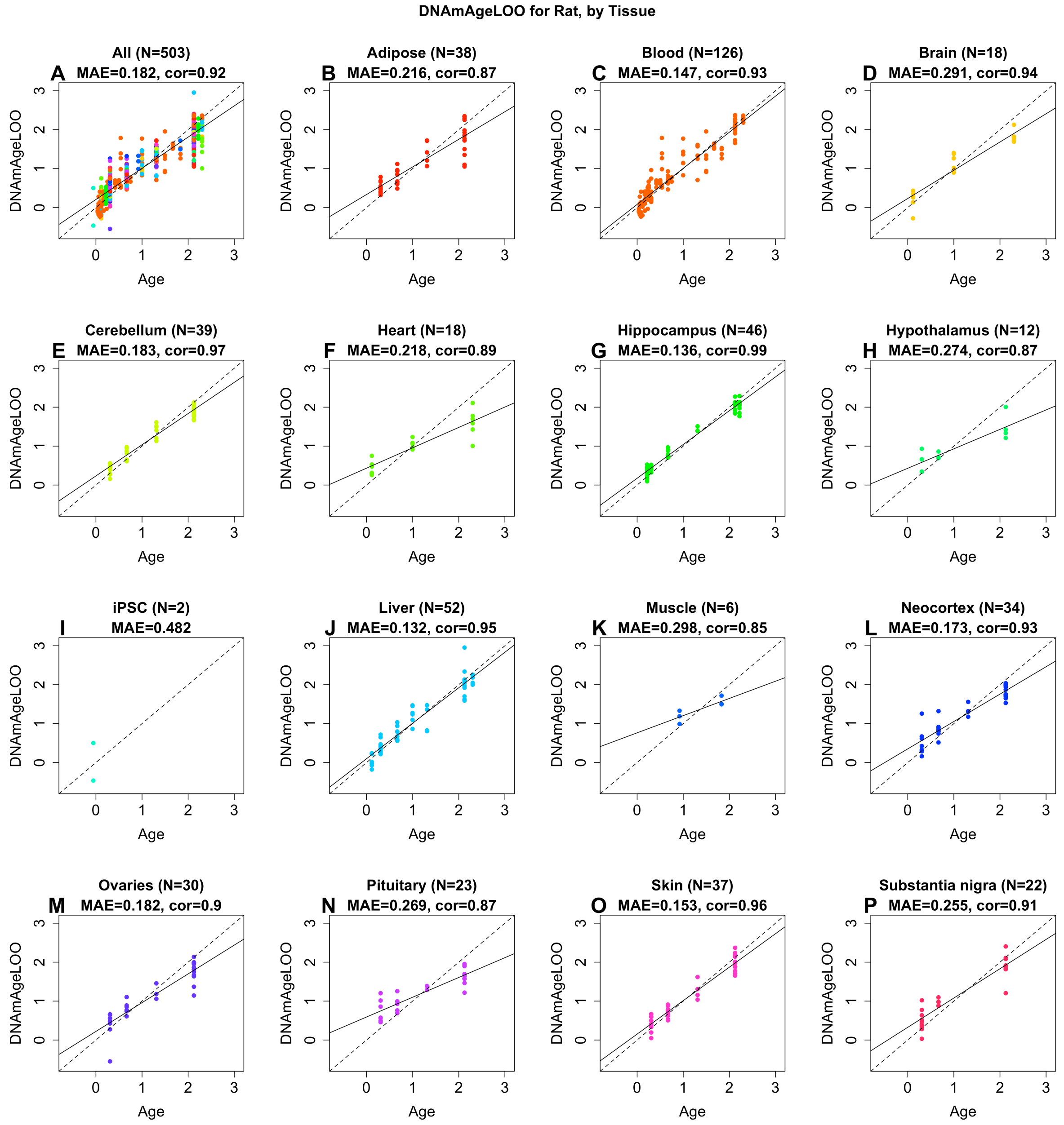

Supplementary Figure 2. Pan tissue clock for rats applied to different tissues. A) All tissues. B) adipose, C) blood, D) whole brain, E) cerebellum, F) heart, G) hippocampus, H) hypothalamus, I) liver, J) brain neocortex, K) ovaries, L) pituitary, M) skin, N) substantia nigra. The title of each panel reports the tissue, sample size, Pearson correlation coefficient and median absolute deviation (median error). Chronological age (x-axis) versus leave-one-sample-out estimate of age.

bioRxiv preprint doi: https://doi.org/10.1101/2023.08.06.552148; this version posted August 7, 2023. The copyright holder for this preprint (which was not certified by peer review) is the author/funder, who has granted bioRxiv a license to display the preprint in perpetuity. It is made available under aCC-BY 4.0 International license.

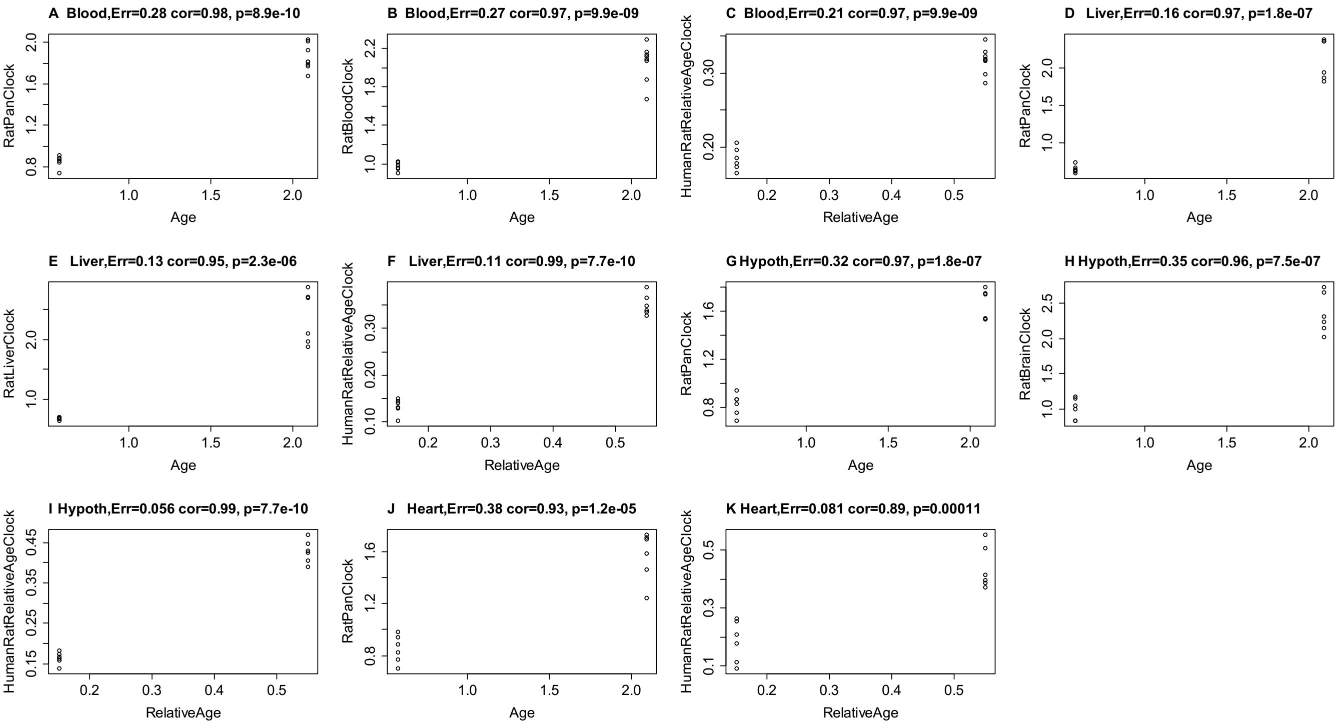

Supplementary Figure 3: Epigenetic clocks applied to independent test data. The six epigenetic clocks were applied to DNA profiles from un-treated samples from the E5 plasma fraction study. The y-axis reports epigenetic age (in years) as measured by the indicated clocks. The heading reports the tissue type, the correlation between epigenetic and chronological age and the median error (in years). While the age correlations are high, the median errors are sub-optimal, up to 0.38 years (panel J). As such, the final versions of the clocks incorporated un-treated samples from the test data as well (Methods).

bioRxiv preprint doi: https://doi.org/10.1101/2023.08.06.552148; this version posted August 7, 2023. The copyright holder for this preprint (which was not certified by peer review) is the author/funder, who has granted bioRxiv a license to display the preprint in perpetuity. It is made available under aCC-BY 4.0 International license.

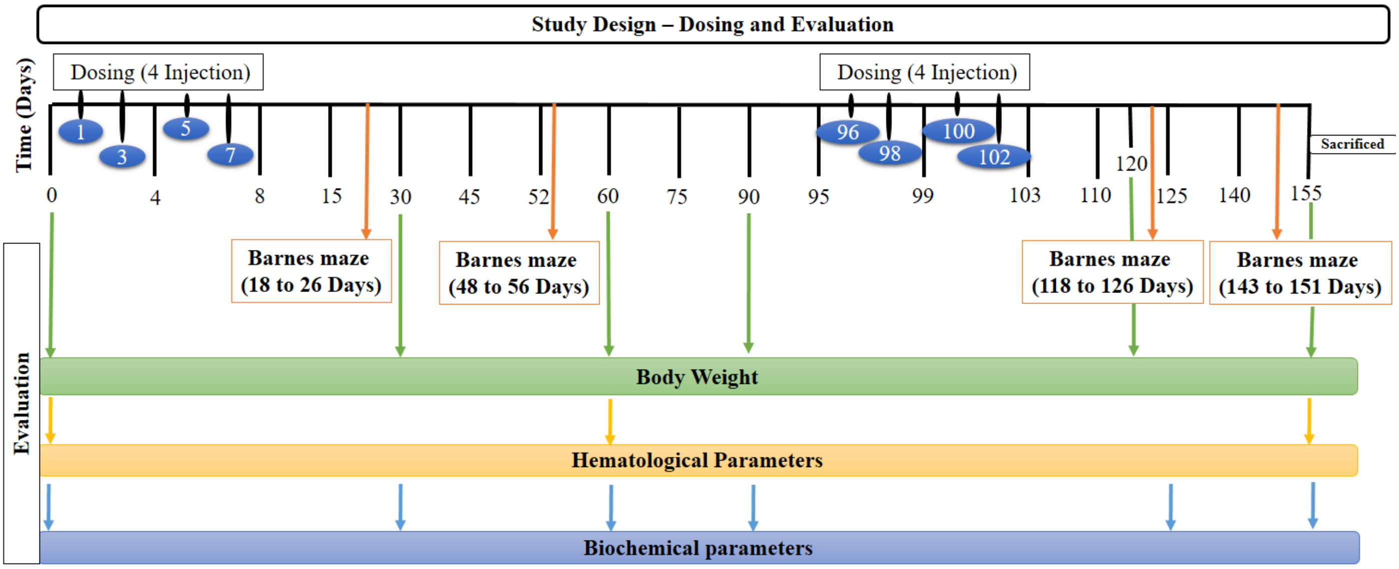

- **TNF alpha and IL-6 levels were measured at all time points.**
- **Antioxidant parameters, Nrf2, Histopathology, SA-β-gal and Oil Red O staining were performed on vital organs after sacrificing animals on 155th day of study.**

Supplementary Figure 4: Schematic representation of the study design with timeline indicating plasma fraction treatments as well as cognitive, physical, haematological and biochemical tests.

bioRxiv preprint doi: https://doi.org/10.1101/2023.08.06.552148; this version posted August 7, 2023. The copyright holder for this preprint (which was not certified by peer review) is the author/funder, who has granted bioRxiv a license to display the preprint in perpetuity. It is made available under aCC-BY 4.0 International license.

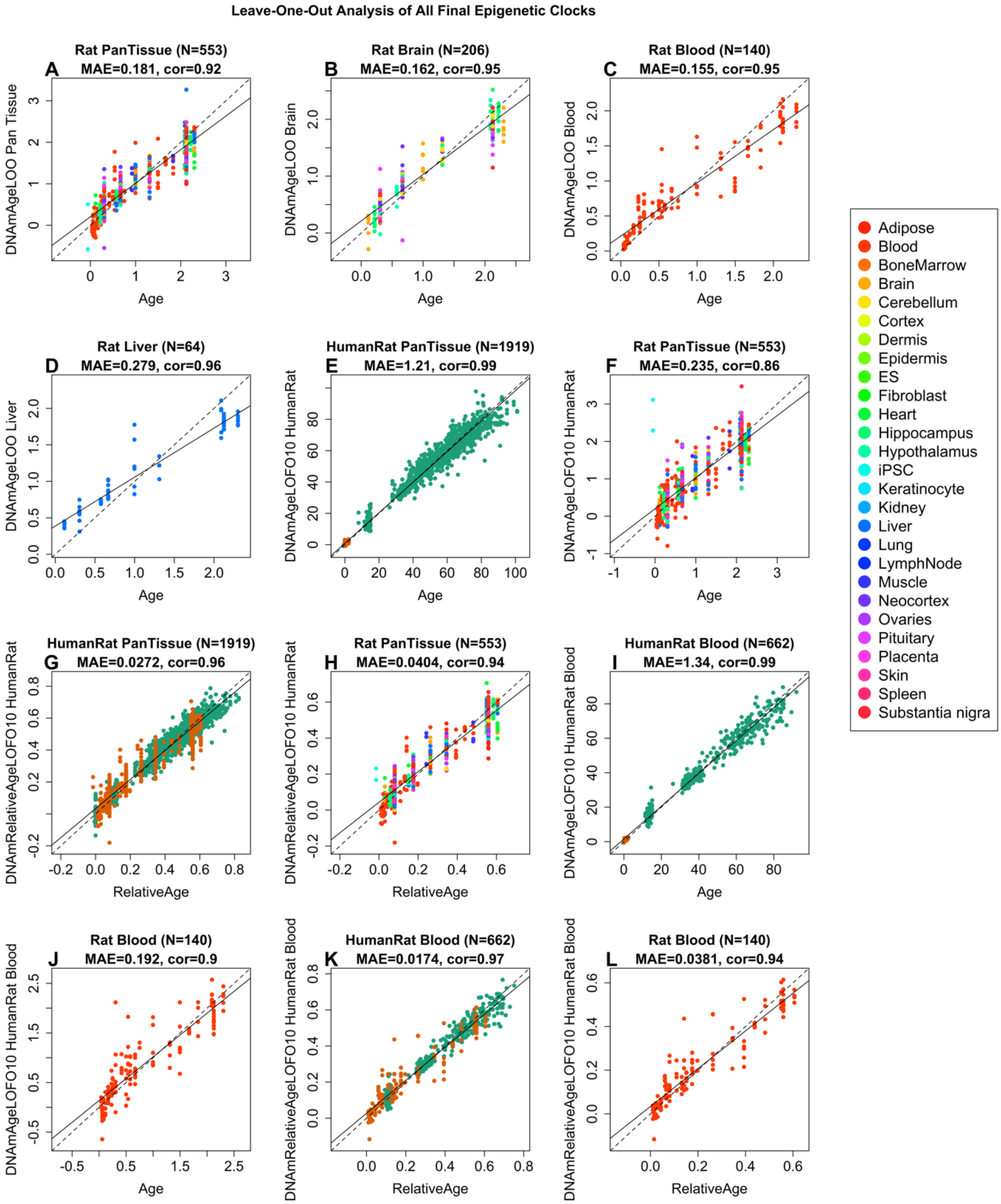

Supplementary Figure 5: This figure is analogous to Figure 1, but it reports estimates of the predictive accuracy of the final version of the rat clocks. Cross-validation was carried out on an increased number of rat tissues (n=553) by combining the original training data (n=503) with rat tissues from untreated animals of the second test data set.

bioRxiv preprint doi: https://doi.org/10.1101/2023.08.06.552148; this version posted August 7, 2023. The copyright holder for this preprint (which was not certified by peer review) is the author/funder, who has granted bioRxiv a license to display the preprint in perpetuity. It is made available under aCC-BY 4.0 International license.

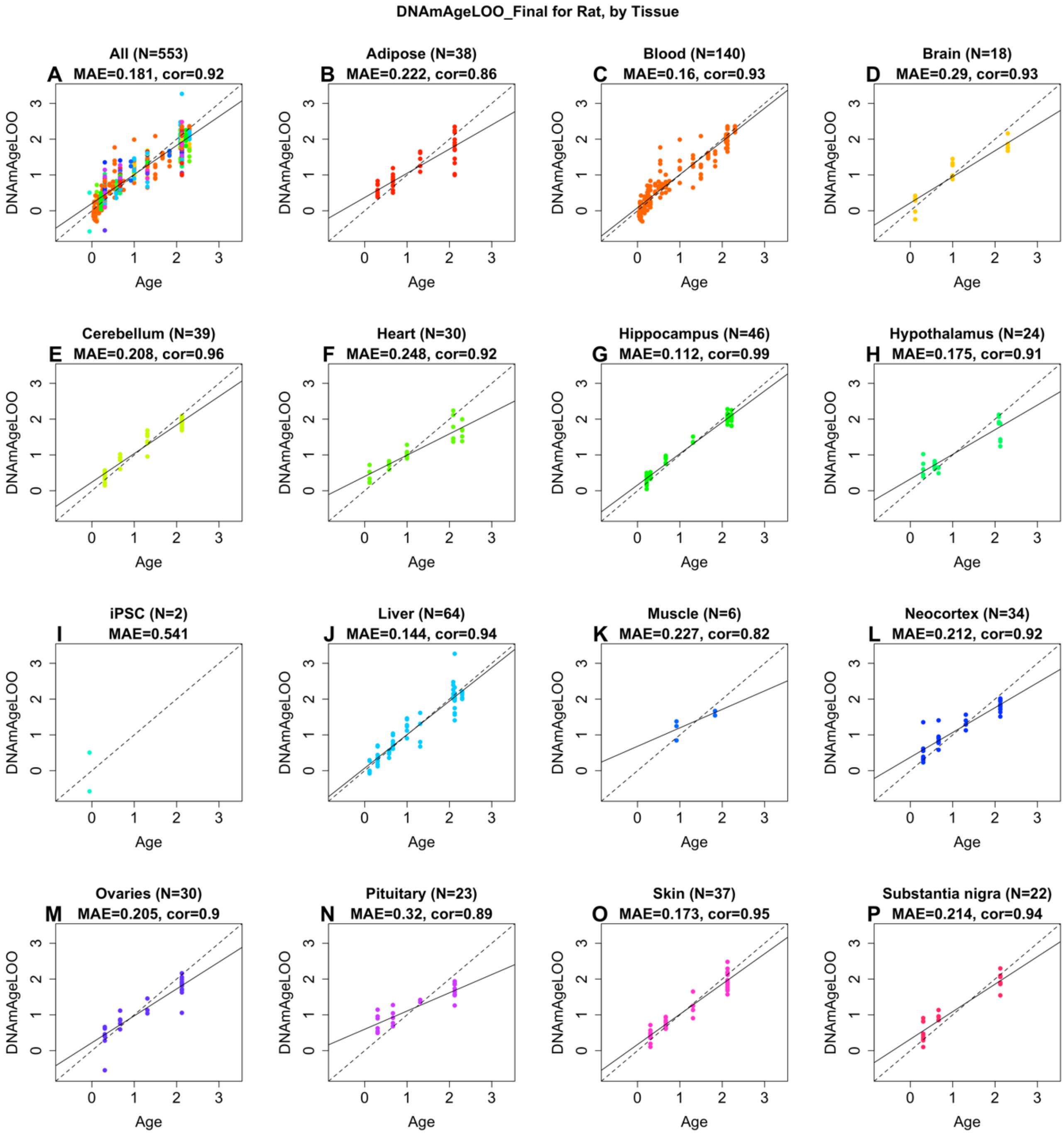

Supplementary Figure 6: Estimating the accuracy of the final version of the pan tissue rat clock. Each panel corresponds to a different source of DNA in rat tissue. Leave one out cross validation estimates of DNAmAge (y-axis) versus chronological age.

bioRxiv preprint doi: https://doi.org/10.1101/2023.08.06.552148; this version posted August 7, 2023. The copyright holder for this preprint (which was not certified by peer review) is the author/funder, who has granted bioRxiv a license to display the preprint in perpetuity. It is made available under aCC-BY 4.0 International license.

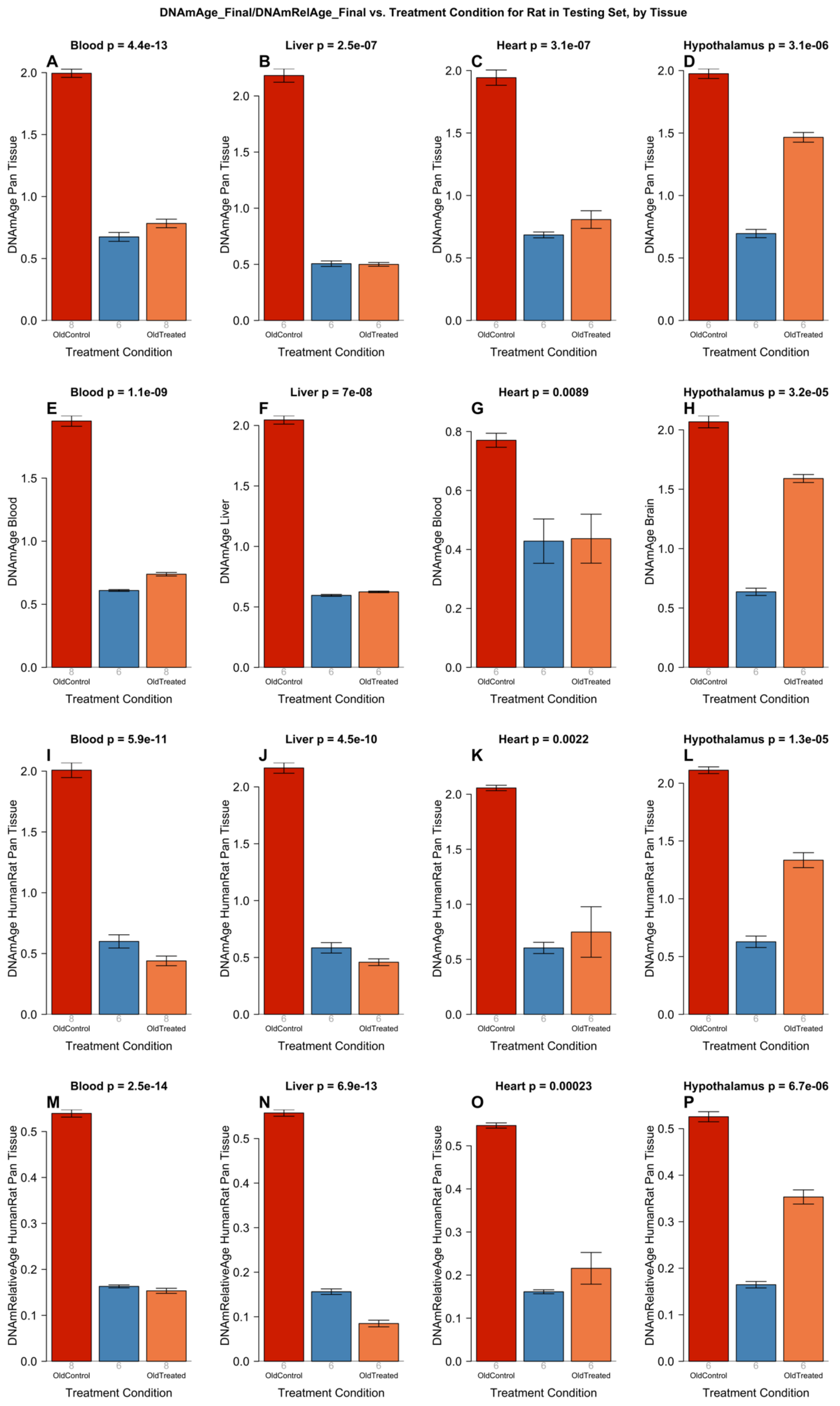

bioRxiv preprint doi: https://doi.org/10.1101/2023.08.06.552148; this version posted August 7, 2023. The copyright holder for this preprint (which was not certified by peer review) is the author/funder, who has granted bioRxiv a license to display the preprint in perpetuity. It is made available under aCC-BY 4.0 International license.

Supplementary Figure 7. **Epigenetic clock analysis of plasma fraction treatment based on the final version of epigenetic clocks.** This figure is analogous to Figure 2, but it reports results for the final version of the epigenetic clock based on an increased number of rat tissues (n=567) (resulting from combining the original n=517 original training data with rat tissues from untreated animals of the test data set). Six epigenetic clocks applied to independent test data from four rat tissue type (columns): blood, liver, heart, and hypothalamus. A-D) Rat pan-tissue clock. E) Rat blood clock applied to blood. F) Rat liver clock applied to liver. G) Rat blood clock applied to heart. H) Rat brain clock applied to hypothalamus. I-L) Human-rat clock measure of absolute age. M-P) Human-rat clock measure of relative age defined as age/maximum species lifespan. Each bar-plot reports the mean value and one standard error. Student T-test p values result from a 2-group comparison of old controls (left bar) versus old treated samples (right bar), i.e. the young controls were omitted.

bioRxiv preprint doi: https://doi.org/10.1101/2023.08.06.552148; this version posted August 7, 2023. The copyright holder for this preprint (which was not certified by peer review) is the author/funder, who has granted bioRxiv a license to display the preprint in perpetuity. It is made available under aCC-BY 4.0 International license.

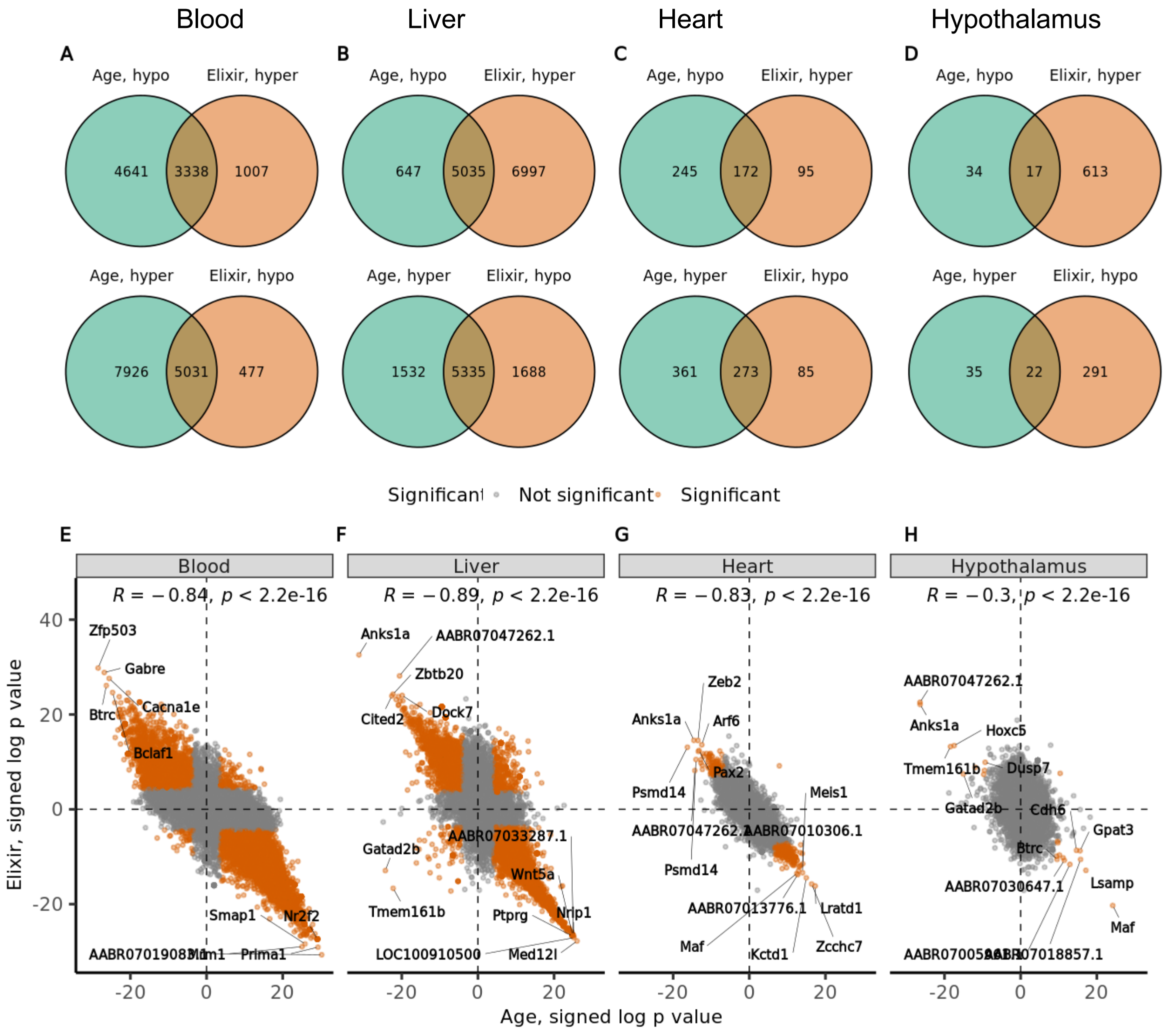

Supplementary Figure 8 (version 1): EWAS results for age versus EWAS results of plasma fraction treatment by tissue type. This figure portrays two distinct versions of the EWAS study, differentiated by their respective definitions of aging effects.

In Version 1, age effects are determined by comparing old control rats to young ones within the test dataset. This analysis may carry potential bias, as samples from old control animals are employed in assessing both age effects (x-axis) and treatment effects (y-axis). To mitigate this concern, Version 2 of the figure (below) illustrates an alternative approach: age effects are calculated exclusively using independent controls, thus ensuring a more unbiased analysis. The Venn diagrams in version 1 indicate the overlaps of significantly differentially hyper- or hypo-methylated aging and E5 affected CpGs across multiple tissue types: blood (A), liver (B), heart (C), and hypothalamus (D). We used a false discovery rate threshold of 0.05 for both aging and treatment effects (Methods). E-H) Each dot corresponds to a CpG on the mammalian array. Aging effects (x-axis) versus E5 rejuvenation effects (y-axis) in specific rat tissue: blood (E), liver (F), heart (G), and hypothalamus (H). Signed P values were defined as follows: sign(FC) * log(p value). Orange color indicates genes with significant differential methylation in both comparisons. Positive/negative values correspond to gain/loss of methylation associated with the treatment.

bioRxiv preprint doi: https://doi.org/10.1101/2023.08.06.552148; this version posted August 7, 2023. The copyright holder for this preprint (which was not certified by peer review) is the author/funder, who has granted bioRxiv a license to display the preprint in perpetuity. It is made available under aCC-BY 4.0 International license.

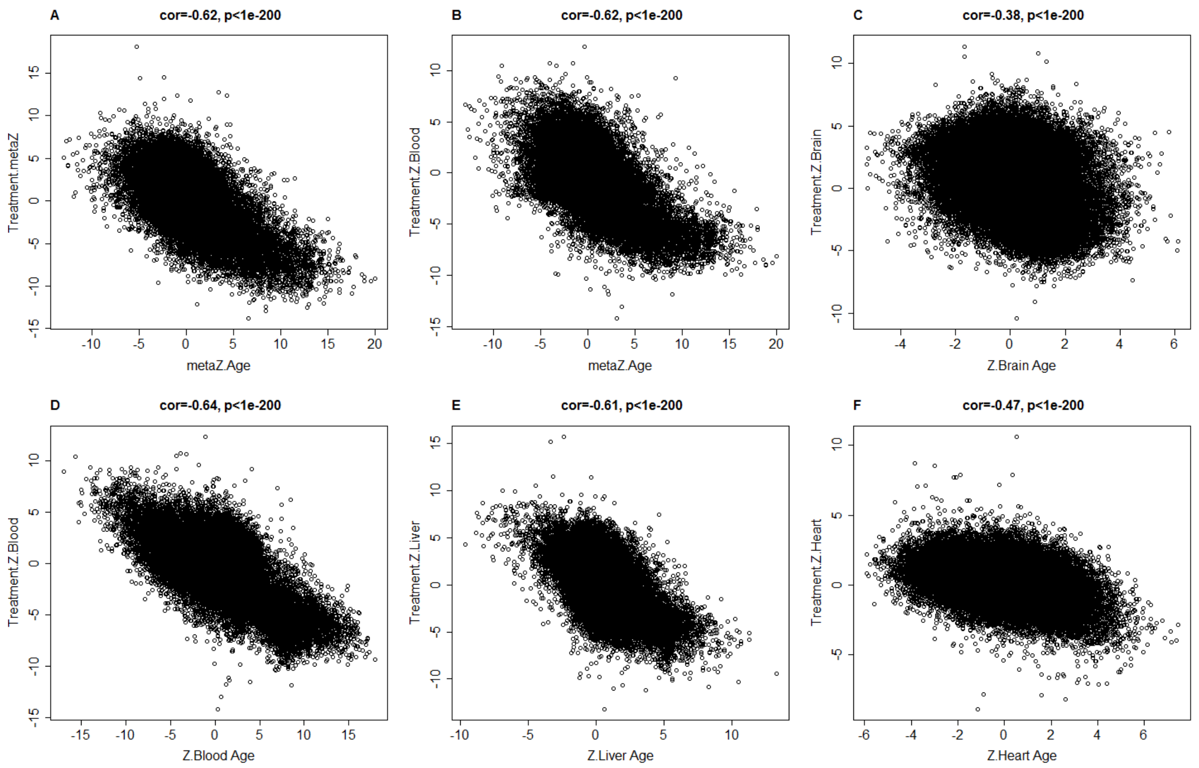

Supplementary Figure 8 (version 2): EWAS results for age versus EWAS results of plasma fraction treatment. This version of our EWAS study is less biased than version 1. Each dot corresponds to a CpG on the mammalian array. The x axis reports Z statistics from a correlation test of CpG methylation versus chronological age (R function "standardScreeningNumericTrait" in the WGCNA package). Positive/negative values correspond to positive/negative correlation coefficients with age, respectively. The y-axis reports a Z statistic for the treatment effect. Positive/negative values correspond to gain/loss of methylation associated with the treatment. A-B) The x axis corresponds to a meta-analysis of age effects across all rat tissues (Adipose, Blood, Brain, Cerebellum, Heart, Hippocampus, Hypothalamus, Liver, Neocortex, Ovaries, Pituitary, Skin, Substantia nigra). Age effects in C) Brain, D) Blood, E) Liver, F) Heart. A) Treatment effects across four tissues. Stouffer's meta-analysis Z statistic across hypothalamus, blood, liver, heart. Treatment effects in individual tissues B) Blood, C) Hypothalamus, D) Blood, E) Liver, F) Heart.

bioRxiv preprint doi: https://doi.org/10.1101/2023.08.06.552148; this version posted August 7, 2023. The copyright holder for this preprint (which was not certified by peer review) is the author/funder, who has granted bioRxiv a license to display the preprint in perpetuity. It is made available under aCC-BY 4.0 International license.

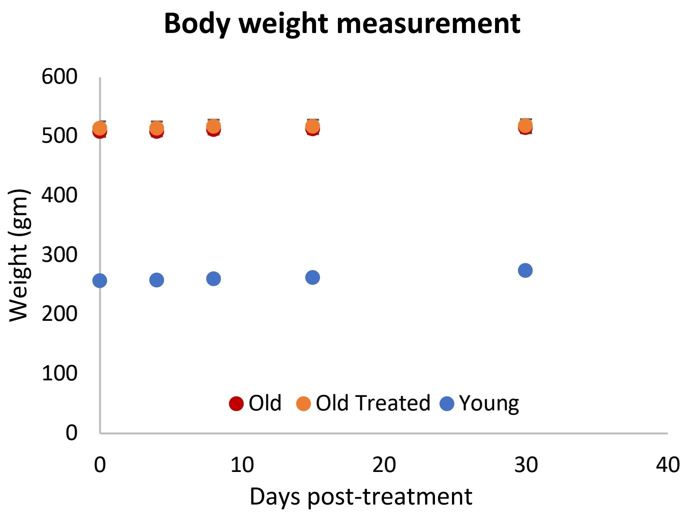

Supplementary Figure 9A. Physical measurements of rats. Weight of rats measured at regular intervals at indicated times during the 155-day experiment. Each group measurement was from 6 rats. The plotted data points represent average values from 6 rats each, with standard error of the mean.

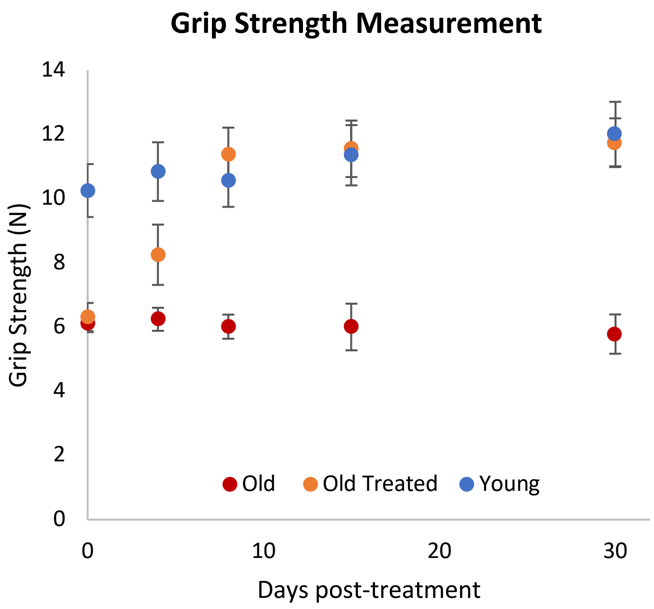

Supplementary Figure 9B. Measurement of grip strength of the indicated rat groups at various times post-treatment. Each group consists of 6 rats. The plotted data points represent average values from 6 rats each, with corresponding 2 standard errors around the mean. Detailed measurements of each parameter are provided in Supplementary Table S5.

bioRxiv preprint doi: https://doi.org/10.1101/2023.08.06.552148; this version posted August 7, 2023. The copyright holder for this preprint (which was not certified by peer review) is the author/funder, who has granted bioRxiv a license to display the preprint in perpetuity. It is made available under aCC-BY 4.0 International license.

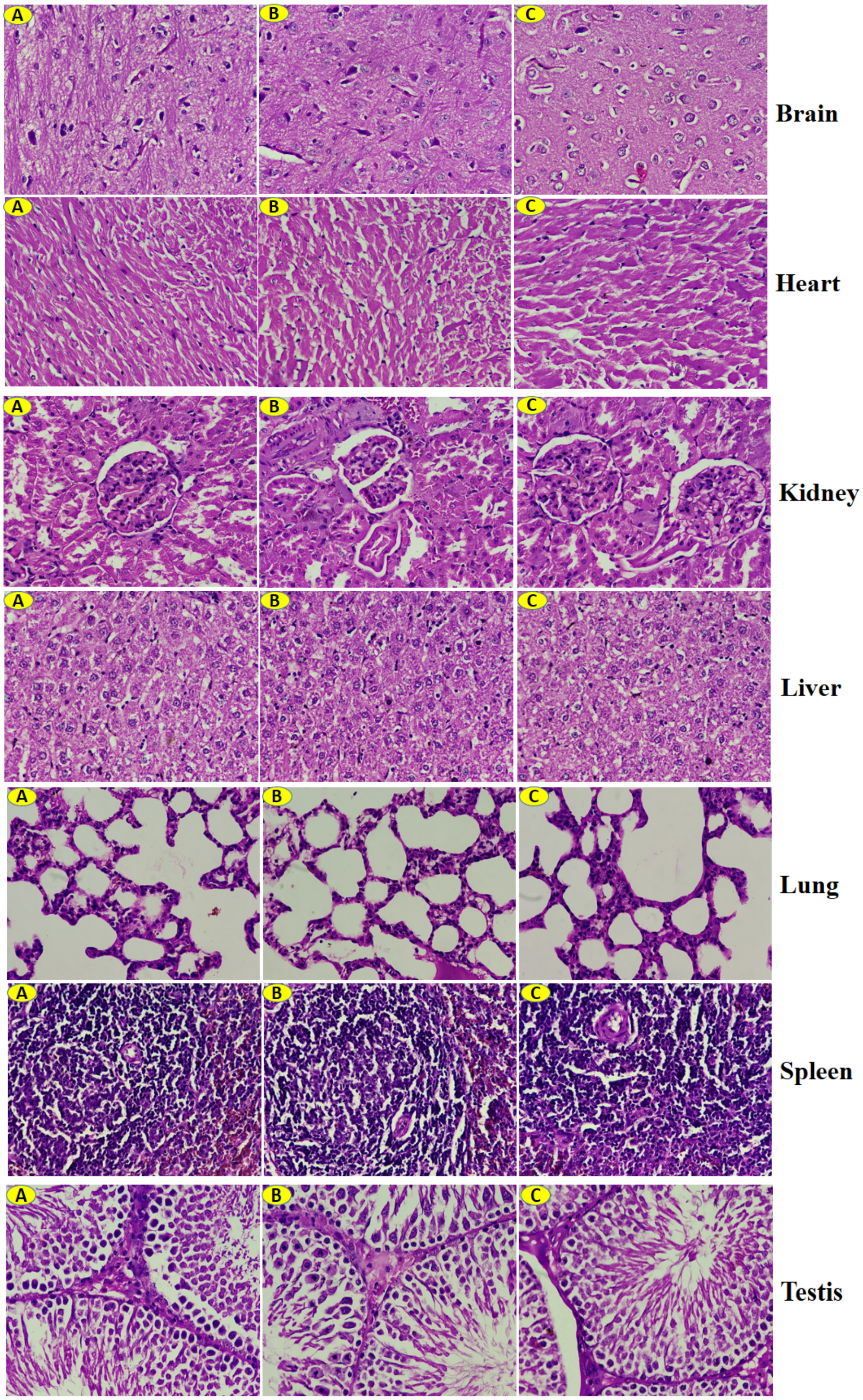

Supplementary Figure 10: Histological analyses of vital organs and tissues of rats employed in experiment. Images of the left column (A) are from old rats; the right column

bioRxiv preprint doi: https://doi.org/10.1101/2023.08.06.552148; this version posted August 7, 2023. The copyright holder for this preprint (which was not certified by peer review) is the author/funder, who has granted bioRxiv a license to display the preprint in perpetuity. It is made available under aCC-BY 4.0 International license.

(C) are images of tissues from young rats while the middle column (B) are images of tissues from old rats treated with plasma fraction. Results of histopathological examination are tabulated in Supplementary Table S6

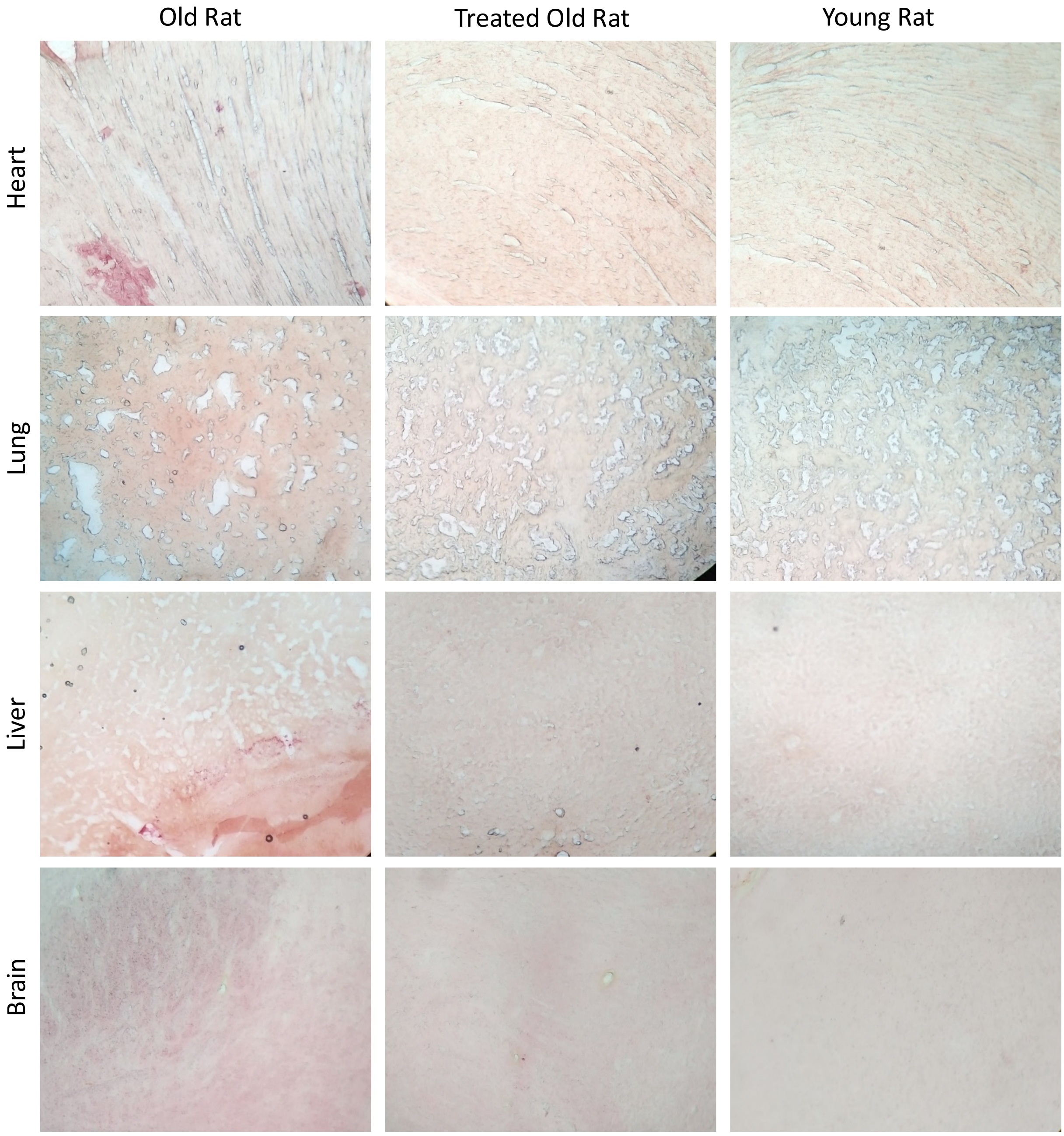

Supplementary Figure 11: Oil Red O staining. Tissues from old untreated rats, Plasma fraction-treated old rats and young rats were subjected to Oil Red O staining to reveal accumulation of fat in tissues.

bioRxiv preprint doi: https://doi.org/10.1101/2023.08.06.552148; this version posted August 7, 2023. The copyright holder for this preprint (which was not certified by peer review) is the author/funder, who has granted bioRxiv a license to display the preprint in perpetuity. It is made available under aCC-BY 4.0 International license.

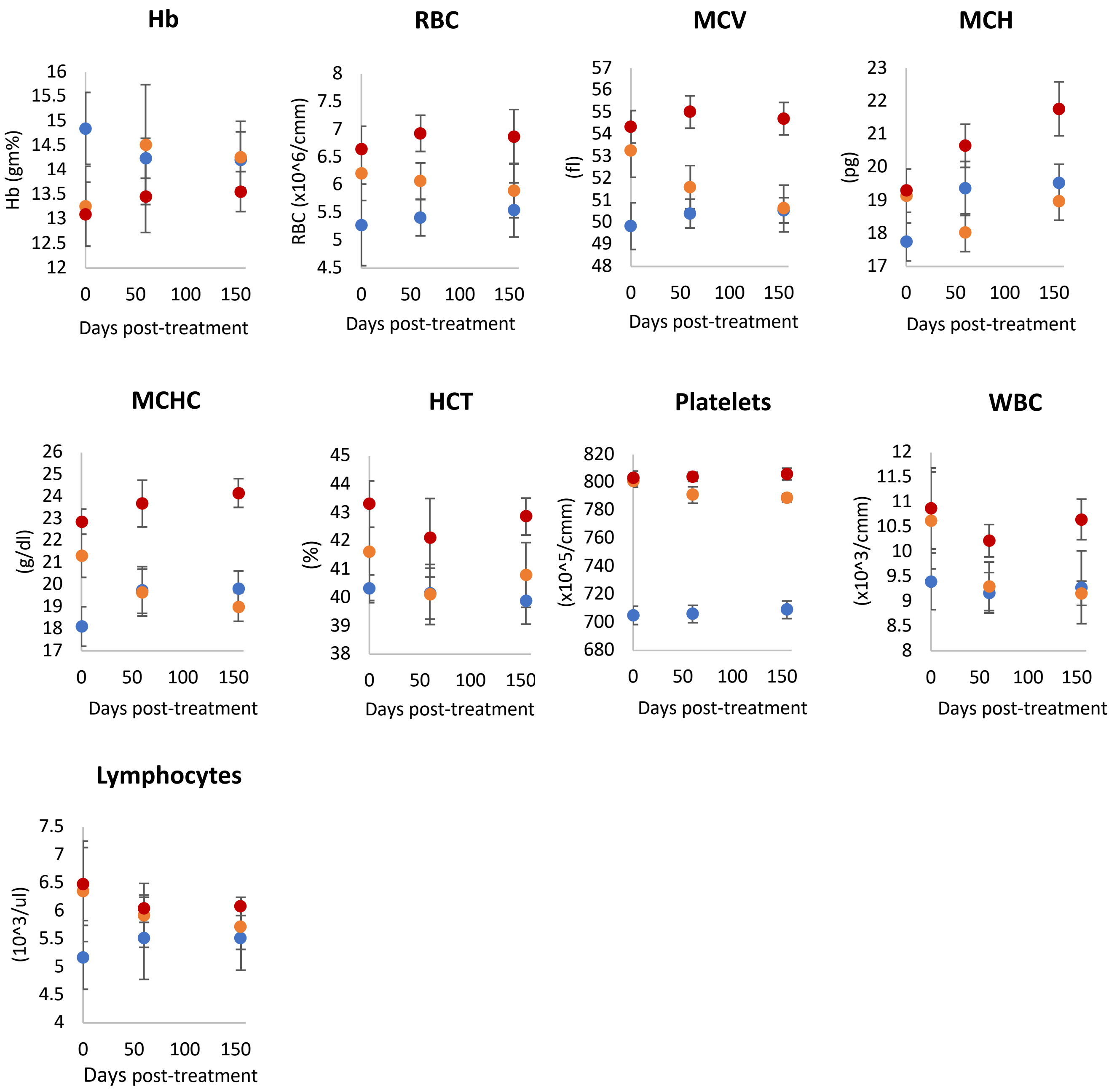

Supplementary Figure 12: Haematological properties. Effects of plasma fraction treatment on haematological properties of rats at 0, 60 and 155 days from the start of experiment. Hb = haemoglobin, RBC = red blood cell count, MCV = mean corpuscular volume, MCH = mean corpuscular haemoglobin, MCHC = mean corpuscular haemoglobin concentration, HCT = haematocrit and WBC = white blood cells. Red dots represent data points of old rats, orange dots represent treated old rats and blue represents young rats. The plotted data points represent average values from 6 rats each, with 2 standard errors around the mean. Detailed measurements of each parameter are provided in Supplementary Table S7.

bioRxiv preprint doi: https://doi.org/10.1101/2023.08.06.552148; this version posted August 7, 2023. The copyright holder for this preprint (which was not certified by peer review) is the author/funder, who has granted bioRxiv a license to display the preprint in perpetuity. It is made available under aCC-BY 4.0 International license.

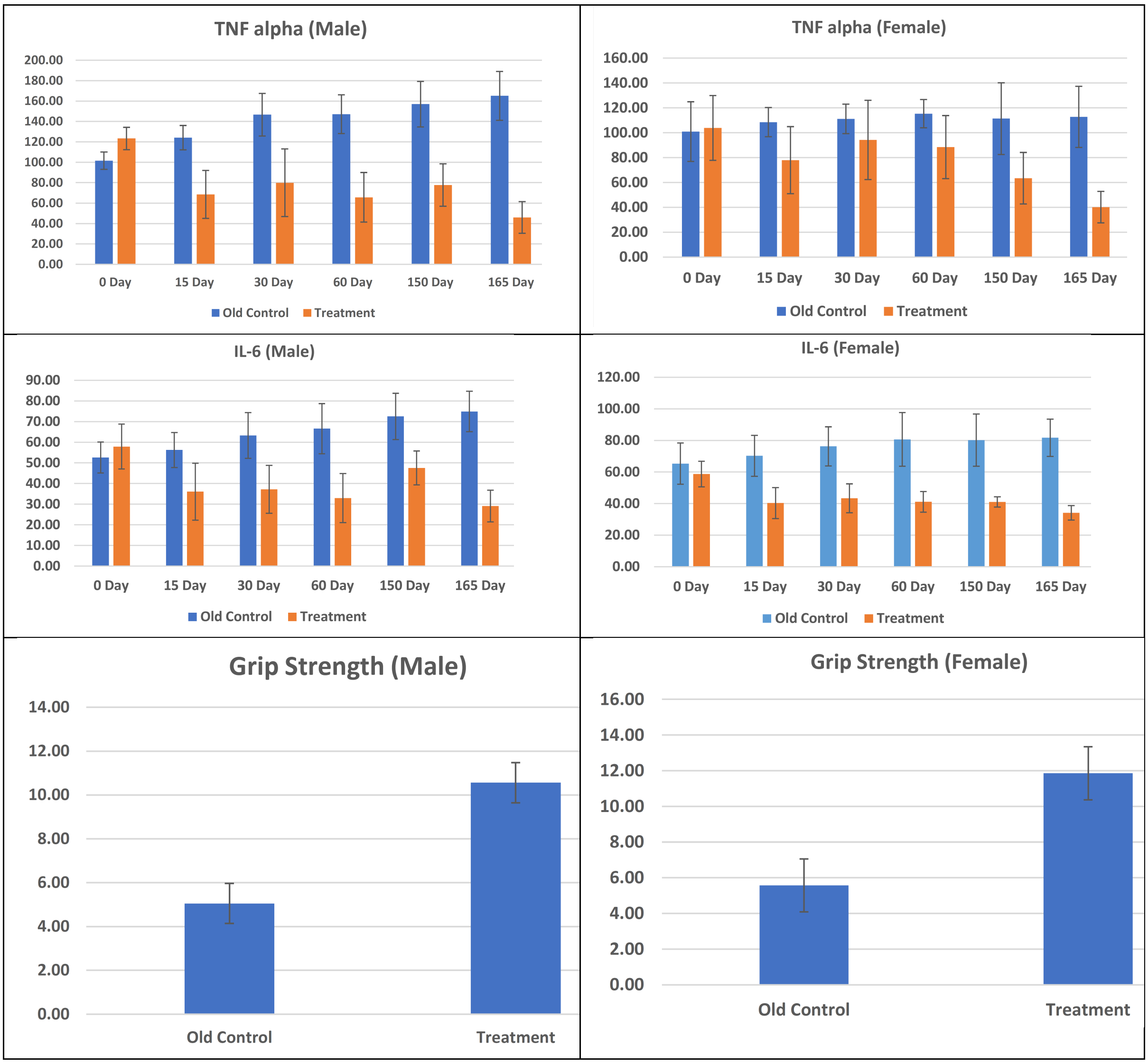

Supplementary Figure 13. Evaluating the effect of E5 in both male and female Sprague Dawley rats. A,B) TNA alpha (y-axis) and C,D) IL-6 are measured in units of picogram/ml. At baseline, the rats were 26 months old. The study analzed 12 male and 12 female rats. Half of the animals were treated with E5. The controls were treated with saline.

bioRxiv preprint doi: https://doi.org/10.1101/2023.08.06.552148; this version posted August 7, 2023. The copyright holder for this preprint (which was not certified by peer review) is the author/funder, who has granted bioRxiv a license to display the preprint in perpetuity. It is made available under aCC-BY 4.0 International license.

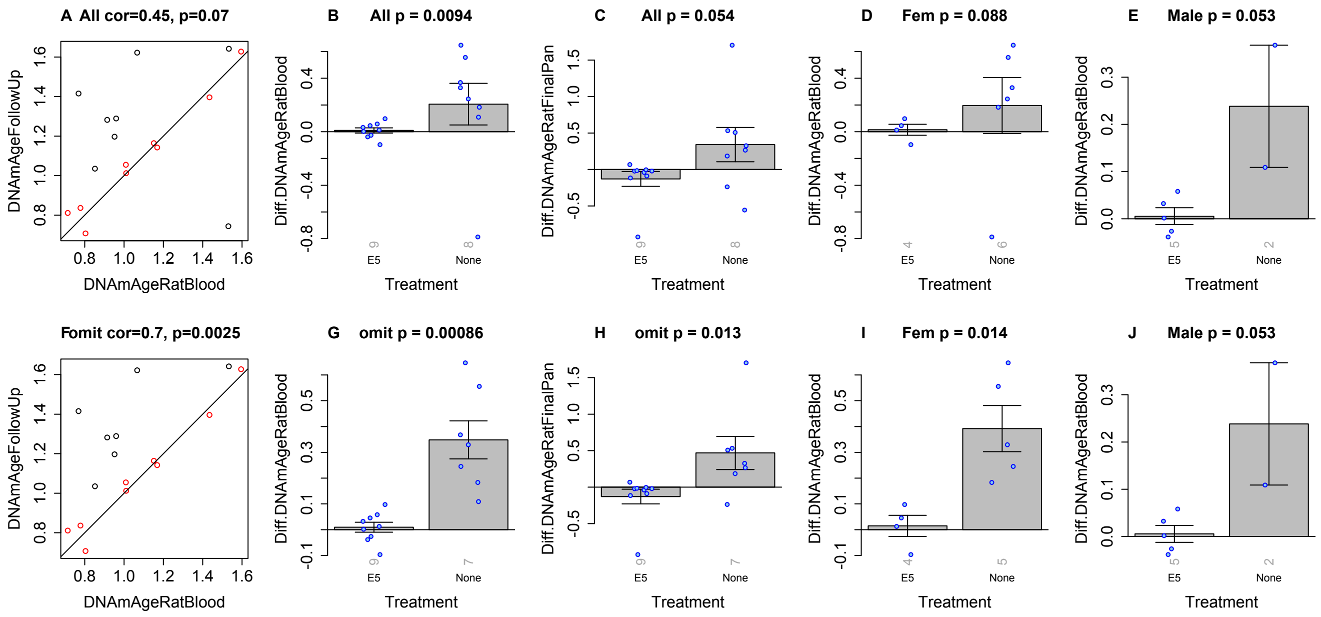

Supplementary Figure 14. Epigenetic clock analysis in blood of male and female rats. This is an independent validation study of epigenetic clocks that is based on a different set of Sprague Dawley rats of both sexes. A) Final version of the rat clock for blood. Baseline measurement (x-axis) versus follow up measurement (15 days after treatment, y-axis). Points (rats) are colored by treatment: red=treated by E5, black=treated with saline only. B,D,E) Difference between follow up measurement and baseline measurement (y-axis) versus treatment status in B) all rats, D) female rats only, E) male rats only. C) is analogous to B) but uses the pan tissue clock for rats.

Panels in the second row (F,G,H,I,J) are analogous to those in the first row but the analysis omitted one control rat (corresponding to the black dot in the lower right of panel A).

The title of the bar plots reports the results of a non-parametric group comparison test (unadjusted two-sided p value from the Kruskal Wallis test). The rotated grey numbers underneath each bar reports the group sizes. Each bar plot reports the mean value and one standard error.

bioRxiv preprint doi: https://doi.org/10.1101/2023.08.06.552148; this version posted August 7, 2023. The copyright holder for this preprint (which was not certified by peer review) is the author/funder, who has granted bioRxiv a license to display the preprint in perpetuity. It is made available under aCC-BY 4.0 International license.

Supplementary Tables

Tissue	N	No. Female	Mean Age	Min. Age	Max. Age
Adipose	38	38	1.2	0.305	2.12
Blood	126	72	0.93	0.0384	2.3
Whole Brain	18	3	1.3	0.115	2.3
Cerebellum	39	39	1.2	0.305	2.12
Heart	18	3	1.3	0.115	2.3
Hippocampus	46	46	1.1	0.216	2.22
Hypothalamus	12	22	1.2	0.305	2.12
iPSC	2	NA	0	0	0
Muscle	6		1.5	1.0	2.0
Liver	52	37	1.2	0.115	2.3
Neocortex	34	34	1.2	0.305	2.12
Ovaries	30	30	1.2	0.305	2.12
Pituitary	23	23	1.2	0.305	2.12
Skin	37	37	1.2	0.305	2.12
Substantia nigra	22	22	1.1	0.305	2.12

Supplementary Table S1. Description of biological materials for training the preliminary epigenetic clocks for rats (N=503). Other supplementary tables provide details on the test data. N=Total number of tissues. Number of females. Age: mean, minimum and maximum in units of years.

Sr. No	Age in Weeks	Age in Months	Number of Animal gender-wise (n=6)	Blood sample	Tissue samples	Total Samples
1	3	0.69	3- Male and 3- Female	6	-	6
2	6	1.38	3- Male and 3- Female	6	Heart, Liver, Brain (18 samples)	24
3	12	2.76	3- Male and 3- Female	6	-	6
4	28	6.44	6- Male	6	-	6
5	52	12.0	6- Male	6	Heart, Liver, Brain (18 samples)	24
6	78	18.0	3- Male and 3- Female	6		6
7	120	27.6	6- Male	6	Heart, Liver, Brain (18 samples)	24
**Total samples (42+54=96)**				**42**	**54**	**96**

Supplementary Table S2. Description of the Sprague Dawley rat samples provided by Dr Kavita Singh for training of rat clock.

bioRxiv preprint doi: https://doi.org/10.1101/2023.08.06.552148; this version posted August 7, 2023. The copyright holder for this preprint (which was not certified by peer review) is the author/funder, who has granted bioRxiv a license to display the preprint in perpetuity. It is made available under aCC-BY 4.0 International license.

Experiment	Group	Age in Weeks	Age in Months	Number of Animal (n=6)	Blood sample	Tissue samples	Total Samples
1	Old Control	109	25.0	6 Male	6+2 replicates	Heart, Liver, Hypothalamus (6 samples each)	26
1	Old Treatment	109	25.0	6 Male	6+2 replicates	Heart, Liver, Hypothalamus (6 samples each)	26
1	Young Control	30	6.9	6 Male	6	Heart, Liver, Hypothalamus (6 samples each)	24
	**Total samples (22+54=76)**				**22**	**54**	**76**

Experiment	Group	Age in Weeks	Age in Months	Number of Animal	Blood sample	Samples	Total Samples
2	Old Control	113.4	26.0	9 (5F, 4M)	2 per animal	Blood draws separated by 2 weeks	18
2	Old Treated	113.4	26.0	8 (6F,2M)	2 per animal	Blood draws separated by 2 weeks	16
	**Total samples**						**34**

Supplementary Table S3. Description of the Sprague Dawley rats employed for testing the effects of the E5 plasma fraction treatment. The two set of independent experiments involved different sets of animals. Experiment 1 profiled multiple organs and tissues from male rats. Experiment 2 analyzed blood methylation data from both male and female rats. Experiment 2 was a longitudinal study based on two blood draws: baseline (before any treatment) and 2 weeks post treatment (E5 treatment or saline control).

bioRxiv preprint doi: https://doi.org/10.1101/2023.08.06.552148; this version posted August 7, 2023. The copyright holder for this preprint (which was not certified by peer review) is the author/funder, who has granted bioRxiv a license to display the preprint in perpetuity. It is made available under aCC-BY 4.0 International license.

Figure	Tissue	Clock	Old Ctrl	Young Ctrl	Old Treated	Perc. Rejuv.	Ave. Rejuv. By Tissue
Fig2B	Liver	DNAmAge Pan Tissue	2.1322	0.5260	0.5715	73.20%	
Fig2F	Liver	DNAmAge Liver	2.4875	0.7220	0.7809	68.61%	
Fig2J	Liver	DNAmAge HumanRat Pan Tissue	4.2965	1.0316	0.9394	78.14%	
Fig2N	Liver	DNAmRelativeAge HumanRat Pan Tissue	0.6752	0.2149	0.1444	78.62%	74.64%
SuppFig12B	Liver	DNAmAge Pan Tissue	2.1818	0.5057	0.4997	77.10%	
SuppFig12F	Liver	DNAmAge Liver	2.0457	0.5955	0.6243	69.48%	
SuppFig12J	Liver	DNAmAge HumanRat Pan Tissue	2.1661	0.5847	0.4583	78.84%	
SuppFig12N	Liver	DNAmRelativeAge HumanRat Pan Tissue	0.5575	0.1562	0.0848	84.79%	77.55%
Fig2D	Hypothalamus	DNAmAge Pan Tissue	1.6158	0.6597	1.2900	20.16%	
Fig2H	Hypothalamus	DNAmAge Brain	2.3387	0.9713	1.9615	16.13%	
Fig2L	Hypothalamus	DNAmAge HumanRat Pan Tissue	2.1391	0.7041	1.4740	31.09%	
Fig2P	Hypothalamus	DNAmRelativeAge HumanRat Pan Tissue	0.4861	0.1879	0.3392	30.22%	24.40%
SuppFig12D	Hypothalamus	DNAmAge Pan Tissue	1.9749	0.6954	1.4656	25.79%	
SuppFig12H	Hypothalamus	DNAmAge Brain	2.0678	0.6363	1.5903	23.09%	
SuppFig12L	Hypothalamus	DNAmAge HumanRat Pan Tissue	2.1123	0.6277	1.3340	36.84%	
SuppFig12P	Hypothalamus	DNAmRelativeAge HumanRat Pan Tissue	0.5256	0.1646	0.3532	32.81%	29.64%
Fig2C	Heart	DNAmAge Pan Tissue	1.6065	0.8285	0.8086	49.66%	
Fig2G	Heart	DNAmAge Blood	0.7898	0.4014	0.4133	47.67%	
Fig2K	Heart	DNAmAge HumanRat Pan Tissue	3.8106	1.7115	1.9973	47.59%	

bioRxiv preprint doi: https://doi.org/10.1101/2023.08.06.552148; this version posted August 7, 2023. The copyright holder for this preprint (which was not certified by peer review) is the author/funder, who has granted bioRxiv a license to display the preprint in perpetuity. It is made available under aCC-BY 4.0 International license.

Fig2O	Heart	DNAmRelativeAge HumanRat Pan Tissue	0.5783	0.2598	0.3409	41.06%	46.49%
SuppFig12C	Heart	DNAmAge Pan Tissue	1.9428	0.6844	0.8074	58.44%	
SuppFig12G	Heart	DNAmAge Blood	0.7704	0.4283	0.4367	43.32%	
SuppFig12K	Heart	DNAmAge HumanRat Pan Tissue	2.0572	0.6036	0.7483	63.62%	
SuppFig12O	Heart	DNAmRelativeAge HumanRat Pan Tissue	0.5471	0.1613	0.2157	60.57%	56.49%
Fig2A	Blood	DNAmAge Pan Tissue	1.7767	0.7497	0.8435	52.53%	
Fig2E	Blood	DNAmAge Blood	2.1596	0.6614	0.7867	63.57%	
Fig2I	Blood	DNAmAge HumanRat Pan Tissue	1.7369	0.6152	0.4429	74.50%	
Fig2M	Blood	DNAmRelativeAge HumanRat Pan Tissue	0.4724	0.1777	0.1585	66.46%	64.26%
SuppFig12A	Blood	DNAmAge Pan Tissue	1.9947	0.6744	0.7830	60.75%	
SuppFig12E	Blood	DNAmAge Blood	1.9523	0.6094	0.7385	62.18%	
SuppFig12I	Blood	DNAmAge HumanRat Pan Tissue	2.0081	0.5998	0.4395	78.11%	
SuppFig12M	Blood	DNAmRelativeAge HumanRat Pan Tissue	0.5394	0.1630	0.1533	71.58%	68.15%

Supplementary Table S4: Mean values of the DNAm age estimates of the six epigenetic clocks in the E5 plasma treatment study. The columns report the figure panels of the corresponding barplot. The tissue that was used to evaluate the clock. The type of clock. The percentage of rejuvenation (last column) was calculated as follows: 100*(1-Old Treated/OldControl). The results for Figure 2 involved the preliminary versions of the six rat clocks. The results for Supplementary Figure 7 involved the final versions of the six rat clocks as detailed in Methods.

According to the 6 epigenetic clocks, the plasma fraction treatment rejuvenated liver by 74.6% (ranging from 68.6% to 78.6% depending on the clock), blood by 64.3% (ranging from 52.5 to 74.5%), heart by 46.5%, and hypothalamus by 24.4%. The rejuvenation effects are even more pronounced if we use the final versions of our epigenetic clocks: liver 77.6%, blood 68.2%, heart 56.5%, hypothalamus 29.6%. According to the final version of the epigenetic clocks, the average rejuvenation across four tissues was 67.40%.

bioRxiv preprint doi: https://doi.org/10.1101/2023.08.06.552148; this version posted August 7, 2023. The copyright holder for this preprint (which was not certified by peer review) is the author/funder, who has granted bioRxiv a license to display the preprint in perpetuity. It is made available under aCC-BY 4.0 International license.

Groups	Grip Strength in N		
	**Old control**	**Treatment**	**Adult Control**
**0 Day**	6.10±0.32	6.31±0.44	10.25±1.01
**4 Day**	6.24±0.44	8.25±0.94	10.84±1.12
**8 Day**	6.01±0.46	11.38±0.83	10.55±0.99
**15 Day**	6.00±0.89	11.55±0.88	11.35±1.15
**30 Day**	5.78±0.75	11.74±0.76	12.01±1.23

Supplementary Table S5: Measurement (with standard deviations) of grip strength of indicated groups of 6 rats each. These average values formed the graphs in Supplementary Figure 9B.

bioRxiv preprint doi: https://doi.org/10.1101/2023.08.06.552148; this version posted August 7, 2023. The copyright holder for this preprint (which was not certified by peer review) is the author/funder, who has granted bioRxiv a license to display the preprint in perpetuity. It is made available under aCC-BY 4.0 International license.

Histopathological evaluation of rat tissues

Rat group	Liver	Lungs	Kidney	Heart	Spleen	Brian	Testes
**Old Control**	Granular degeneration of mild severity, minimally multifocal minimal periportal mononuclear cell infiltration	NAD	NAD	NAD	NAD	NAD	NAD
**Old Control**	Granular degeneration of mild severity	NAD	NAD	NAD	NAD	NAD	NAD
**Old Control**	Granular degeneration of mild severity	NAD	NAD	NAD	NAD	NAD	NAD
**Treatment**	Granular degeneration of mild severity	NAD	NAD	NAD	NAD	NAD	NAD
**Treatment**	Granular degeneration of mild severity	NAD	NAD	NAD	NAD	NAD	NAD
**Treatment**	Granular degeneration of mild severity	NAD	NAD	NAD	NAD	NAD	NAD
**Young Control**	Granular degeneration of mild severity	NAD	NAD	NAD	NAD	NAD	NAD
**Young Control**	Granular degeneration of mild severity	NAD	NAD	NAD	NAD	NAD	NAD
**Young Control**	Granular degeneration of mild severity	NAD	NAD	NAD	NAD	NAD	NAD

Supplementary Table S6: Results of histopathological analyses of rat tissues. Lesions suggestive of any toxicity were not noted. NAD= No Abnormalities Detected. Representative images of the tissues are shown in Supplementary Figure 9.

bioRxiv preprint doi: https://doi.org/10.1101/2023.08.06.552148; this version posted August 7, 2023. The copyright holder for this preprint (which was not certified by peer review) is the author/funder, who has granted bioRxiv a license to display the preprint in perpetuity. It is made available under aCC-BY 4.0 International license.

	**Groups**	Hb (gm %)	RBC (x 10^6 /cmm)	WBC (X 10^3 /cmm)	Platelets (X 10^5/ cmm)	HCT (%)	MCV (fl)	MCH (pg)	MCHC g/dl	Lymphocytes (10^3 cells/µl)
0 Day	Old	13.10±0.8	6.65±0.5	10.87±1.0	803.00±6.2	43.03±1.0	54.34±0.9	19.29±0.8	22.86±0.7	6.48±0.8
	Old treated	13.26±1.0	6.21±0.6	10.63±1.2	801.83±5.3	41.61±2.1	53.27±1.5	19.13±0.7	21.31±1.2	6.35±1.1
	Young	14.85±0.9	5.28±0.9	9.40±0.7	705.33±8.0	40.31±0.6	49.83±1.3	17.74±1.0	18.10±1.1	5.17±0.7
60 Day	Old	13.46±0.9	6.93±0.4	10.22±0.4	804.17±4.0	42.11±1.7	55.01±0.9	20.65±0.8	23.68±1.3	6.04±0.3
	Old treated	14.52±1.5	6.07±0.4	9.30±0.6	791.67±7.2	40.11±1.3	51.60±1.2	18.02±1.0	19.64±1.3	5.92±0.7
	Young	14.24±0.5	5.41±0.4	9.17±0.5	706.17±7.6	40.14±1.1	50.40±0.8	19.36±0.7	19.76±1.3	5.51±0.9
155 Day	Old	13.56±0.5	6.87±0.6	10.65±0.5	806.50±5.1	42.86±0.8	54.71±0.9	21.77±1.0	24.16±0.8	6.08±0.2
	Old treated	14.26±0.9	5.90±06	9.16±0.3	789.00±3.5	40.80±1.4	50.63±1.3	18.97±0.7	18.99±0.8	5.72±0.5
	Young	14.21±0.7	5.55±0.6	9.28±0.9	709.83±7.7	39.88±1.0	50.55±0.7	19.52±0.7	19.81±1.0	5.51±0.7

Supplementary Table S7: Blood indices measurements (with standard deviation) at indicated time points post-treatment. Each measurement was taken from 6 rats per group. These average values formed the graphs in Supplementary Figure 12.

bioRxiv preprint doi: https://doi.org/10.1101/2023.08.06.552148; this version posted August 7, 2023. The copyright holder for this preprint (which was not certified by peer review) is the author/funder, who has granted bioRxiv a license to display the preprint in perpetuity. It is made available under aCC-BY 4.0 International license.

Sr. No.	Parameter	Group	Old	Old Treated	Young
1	**Total Bilirubin (mg/dL)**	0 Day	0.90±0.10	0.90±0.11	0.56±0.08
		30 Day	0.92±0.09	0.83±0.12	0.55±0.09
		60 Day	0.92±0.11	0.82±0.10	0.57±0.08
		90 Day	0.97±0.11	0.78±0.09	0.57±0.08
		125 Day	1.02±0.12	0.72±0.09	0.59±0.08
		155 Day	**1.08±0.12**	**0.68±0.07 ### ***	**0.60±0.07**
2	**Direct Bilirubin (mg/dL)**	0 Day	0.575±0.06	0.583±0.07	0.257±0.05
		30 Day	0.588±0.06	0.563±0.06	0.258±0.05
		60 Day	0.605±0.07	0.520±0.05	0.273±0.04
		90 Day	0.618±0.06	0.490±0.06	0.283±0.04
		125 Day	0.640±0.06	0.468±0.07	0.293±0.05
		155 Day	**0.680±0.08**	**0.438±0.04 ### *****	**0.308±0.04**
3	**Glucose (mg/dL)**	0 Day	173.0±5.57	174.9±4.97	152.0±8.80
		30 Day	174.4±5.13	169.6±2.88	152.3±8.34
		60 Day	178.7±5.02	167.5±3.53	155.4±8.69
		90 Day	180.0±5.37	165.3±3.00	157.9±9.60
		125 Day	183.2±5.04	163.5±3.59	161.2±9.55
		155 Day	**186.0±3.32**	**163.6±4.10 ### ***	**164.6±10.09**
4	**Triglyceride (mg/dL)**	0 Day	57.2±10.16	56.4±10.45	25.0±9.01
		30 Day	70.5±5.45	45.1±6.67	28.9±8.80
		60 Day	79.3±5.52	45.4±5.84	30.8±7.83
		90 Day	84.1±5.63	43.6±6.05	33.1±7.25
		125 Day	91.4±5.08	41.7±5.78	34.7±7.92
		155 Day	**103.7±5.30**	**38.0±5.64 ###**	**37.9±8.26**
5	**HDL (mg/dL)**	0 Day	109.7±9.19	111.3±8.95	142.5±6.71
		30 Day	110.7±7.05	117.2±8.44	144.3±5.92
		60 Day	108.9±6.89	127.0±8.16	145.1±6.25
		90 Day	108.0±7.67	130.6±9.13	147.2±6.70
		125 Day	105.9±7.78	138.2±10.11	149.0±6.49
		155 Day	**102.3±7.24**	**147.2±8.58 ##****	**151.6±6.68**
6	**Cholesterol (mg/dL)**	0 Day	46.8±6.74	45.9±6.85	17.3±3.17

bioRxiv preprint doi: https://doi.org/10.1101/2023.08.06.552148; this version posted August 7, 2023. The copyright holder for this preprint (which was not certified by peer review) is the author/funder, who has granted bioRxiv a license to display the preprint in perpetuity. It is made available under aCC-BY 4.0 International license.

		30 Day	48.1±6.49	40.3±6.76	17.8±4.21
		60 Day	49.1±6.40	37.6±5.88	18.4±4.33
		90 Day	50.4±5.76	34.9±7.13	19.2±4.12
		125 Day	53.7±5.76	32.0±6.57	21.0±3.78
		155 Day	**56.6±5.78**	**28.1±5.45 ### ****	**23.0±3.75**
**7**	**Creatinine (mg/dL)**	0 Day	1.08±0.10	1.07±0.12	0.29±0.02
		30 Day	1.06±0.09	1.02±0.13	0.35±0.04
		60 Day	1.31±0.24	0.87±0.11	0.39±0.03
		90 Day	1.54±0.26	0.80±0.10	0.44±0.04
		125 Day	1.78±0.22	0.70±0.08	0.50±0.03
		155 Day	**2.03±0.33**	**0.63±0.08 ### ***	**0.54±0.02**
**8**	**BUN (mg/dL)**	0 Day	16.23±1.21	16.19±0.92	4.03±0.13
		30 Day	16.36±1.17	15.26±0.74	4.07±0.15
		60 Day	16.66±1.14	14.57±0.66	4.14±0.15
		90 Day	16.80±1.17	13.27±0.78	4.22±0.15
		125 Day	16.99±1.20	11.05±0.79	4.32±0.16
		155 Day	**17.11±1.22**	**8.94±0.73 ### *****	**4.46±0.15**
**9**	**SGPT (IU/L)**	0 Day	34.30±1.65	34.12±1.91	22.20±1.48
		30 Day	34.06±1.42	32.20±1.52	23.25±1.52
		60 Day	34.93±1.21	30.78±1.41	24.56±1.43
		90 Day	36.02±1.18	29.78±1.03	25.70±1.78
		125 Day	37.16±1.11	28.87±0.89	27.05±1.84
		155 Day	**38.29±1.23**	**28.03±0.76 ### ***	**27.56±1.52**
**10**	**SGOT (IU/L)**	0 Day	86.24±3.77	86.79±2.35	41.53±1.93
		30 Day	90.02±3.95	80.74±2.15	44.86±1.87
		60 Day	92.41±3.69	75.26±2.28	45.39±1.78
		90 Day	94.97±3.87	66.39±3.17	47.55±2.41
		125 Day	96.18±3.33	60.60±1.22	50.39±2.36
		155 Day	**97.45±2.38**	**54.13±1.85 ### ****	**53.54±2.43**
**11**	**Total protein (g/dl)**	0 Day	7.59±1.06	7.75±0.88	4.74±0.95
		30 Day	8.22±1.07	7.55±0.78	5.15±0.73
		60 Day	8.73±0.73	7.39±0.80	5.57±0.77
		90 Day	9.83±0.59	7.15±0.94	5.81±0.87

bioRxiv preprint doi: https://doi.org/10.1101/2023.08.06.552148; this version posted August 7, 2023. The copyright holder for this preprint (which was not certified by peer review) is the author/funder, who has granted bioRxiv a license to display the preprint in perpetuity. It is made available under aCC-BY 4.0 International license.

		125 Day	10.96±0.35	7.04±0.88	6.56±0.80
		155 Day	**12.14±0.53**	**7.12±0.86 ##**	**7.01±0.86**

Supplementary Table S8: Detailed vital organ biomarker measurements of rats at stated time points post-plasma fraction treatment. The measurements (with standard deviation) were from 6 rats per group. These average values formed the graphs in Figure 3.

bioRxiv preprint doi: https://doi.org/10.1101/2023.08.06.552148; this version posted August 7, 2023. The copyright holder for this preprint (which was not certified by peer review) is the author/funder, who has granted bioRxiv a license to display the preprint in perpetuity. It is made available under aCC-BY 4.0 International license.

Glycoform	Treatment effect	Treatment standard error	*p*-value	Adjusted *p*-value
IgG2b-H3N5F1	1.969	0.460	8.29E-05	2.16E-03
IgG2a-H4N4F1	1.484	0.385	3.20E-04	4.16E-03
IgG2a-H3N4F1	-1.561	0.421	5.10E-04	4.42E-03
IgG2a-H5N4F1	1.606	0.452	8.11E-04	5.27E-03
IgG2b-H5N5F1	-1.339	0.419	2.34E-03	1.22E-02
IgG2b-H4N4F1	0.841	0.303	7.49E-03	2.85E-02
IgG2c-H3N5F1	-1.137	0.411	7.66E-03	2.85E-02
IgG2b-H3N3F1	-1.290	0.496	1.18E-02	3.84E-02
IgG2c-H5N4F1G1	-1.182	0.473	1.54E-02	4.46E-02
IgG2b-H3N4F1	-0.802	0.341	2.21E-02	5.75E-02
IgG2a-H5N4F1G1	1.106	0.475	2.65E-02	6.13E-02
IgG2a-H4N5F1G1	1.097	0.477	2.83E-02	6.13E-02
IgG2c-H3N4	-0.958	0.462	4.23E-02	8.45E-02
IgG2c-H3N4F1	0.970	0.480	4.79E-02	8.89E-02
IgG2b-H4N5F1G1	0.856	0.444	5.84E-02	9.66E-02
IgG2b-H5N4F1G1	0.691	0.353	5.95E-02	9.66E-02
IgG2a-H3N5F1	0.788	0.442	7.97E-02	1.22E-01
IgG2a-H4N4F1G1	0.922	0.536	9.01E-02	1.30E-01
IgG2c-H4N4F1G1	-0.777	0.494	1.20E-01	1.64E-01
IgG2b-H5N4F1	-0.534	0.379	1.67E-01	2.17E-01
IgG2a-H8N2	0.503	0.531	3.46E-01	4.28E-01
IgG2c-H3N3F1	0.348	0.383	3.67E-01	4.34E-01
IgG2c-H4N4F1	0.399	0.468	3.95E-01	4.47E-01
IgG2a-H3N4	0.389	0.527	4.62E-01	5.00E-01
IgG2a-H3N3F1	-0.215	0.484	6.58E-01	6.84E-01
IgG2b-H4N4F1G1	0.141	0.448	7.53E-01	7.53E-01

Supplementary Table S9. Glycan age analysis in rat blood. Plasma fraction E5 treatment effect on rat IgG2a, 2b, and 2c N-glycoform composition. Glycoproteomic data was analyzed using a linear mixed-effects model and p-values < 0.05 were considered statistically significant. The rows correspond to different rat IgG tryptic peptides as detailed in the following panel.

Rat IgG tryptic peptides and UniProt accesion numbers:
IgG2a – P20760, QSNSTLR
IgG2b – P20761, EEQYNSTFR
IgG2c – P20762, VFTAQTQPHEEQLNGTFR
N – glycosylation site
Glycan composition:
H – hexose
N – *N*-acetylglucosamine
F – fucose
G – *N*-glycolylneuraminic acid

bioRxiv preprint doi: https://doi.org/10.1101/2023.08.06.552148; this version posted August 7, 2023. The copyright holder for this preprint (which was not certified by peer review) is the author/funder, who has granted bioRxiv a license to display the preprint in perpetuity. It is made available under aCC-BY 4.0 International license.

Technical Details and software for the rat clocks

The epigenetic clock software for rats can be applied to data generated on the mammalian array platform [1]. New data for epigenetic clock studies can be generated with the mammalian methylation array (HorvathMammalMethylChip40), which is distributed by the Epigenetic Clock Development Foundation: https://clockfoundation.org/

The coefficient values and CpGs underlying the clocks can be found in a Supplementary Table.

Statistical methods used for building the clocks

The epigenetic clocks were used by employing a single elastic net regression model analysis (R function glmnet). We use used Leave-one-out analysis (LOO) using a single lambda value. We chose the following parameters for the glmnet R function (Alpha: 0.5, CV Fold: 10, Lambda choice for Clock: 1 standard error above minimum CV-MSE).

We report 2 sets of clocks (preliminary versions and final versions). We recommend to use the final versions. We only report the preliminary versions so that readers can recreate Figure 1 and Figure 2 in our article.

Clocks

1) The preliminary and final version of the pan tissue clock for rats is based on 195 CpGs and 193 CpGs which are specified in columns "Coef.PrelimRatPanTissue" and "Coef.FinalRatPanTissue", respectively. No age transformation was carried out.
2) The preliminary and final version of the blood tissue clock for rats is based on 48 CpGs and 51 CpGs which are specified in columns "Coef.PrelimRatBloodTissue" and "Coef.FinalRatBloodTissue", respectively. No age transformation was carried out.
3) The preliminary and final version of the Brain tissue clock for rats is based on 99 CpGs and 108 CpGs which are specified in columns "Coef.PrelimRatBrainTissue" and "Coef.FinalRatBrainTissue", respectively. No age transformation was carried out.
4) The preliminary and final version of the Liver tissue clock for rats is based on 62 CpGs and 46 CpGs which are specified in columns "Coef.PrelimRatLiverTissue" and "Coef.FinalRatLiverTissue", respectively. No age transformation was carried out.

5) Preliminary and final versions of the human rat pan tissue clock are specified in Coef.PrelimHumanRatPanTissueLogLinearAge (700 CpGs) and Coef.FinalHumanRatPanTissueLogLinearAge (989 CpGs). Note that a log linear transformation was applied.
6) Preliminary and final versions of the human rat blood tissue clock are specified in Coef.PrelimHumanRatBloodTissueLogLinearAge (97 CpGs) and Coef.FinalHumanRatBloodTissueLogLinearAge (72 CpGs). Note that a log linear transformation was applied.
7) Preliminary and final versions of the human rat pan tissue clock for RELATIVE AGE are specified in Coef.PrelimHumanRatRelativeAgePan (870 CpGs) and Coef.FinalHumanRatRelativeAgePan (738 CpGs). Note that we define

bioRxiv preprint doi: https://doi.org/10.1101/2023.08.06.552148; this version posted August 7, 2023. The copyright holder for this preprint (which was not certified by peer review) is the author/funder, who has granted bioRxiv a license to display the preprint in perpetuity. It is made available under aCC-BY 4.0 International license.

RelativeAge=Age/maxLifespan where maxLifespan is 122.5 for humans and 3.8 years for rats, respectively. Age is in units of years.

8) Preliminary and final versions of the human rat blood tissue clock for RELATIVE AGE are specified in Coef.PrelimHumanRatRelativeAgeBlood (138 CpGs) and Coef.FinalHumanRatRelativeAgeBlood (145 CpGs).

The DNAm Age estimate is estimated as follows. Form a weighted linear combination of the CpGs whose details can be found in the Supplementary File, SupplementaryData.RatClockCoef.csv.
This will result in a number referred to as LinearCombination. Some of the clocks involve a log linear transformation whose inverse needs to be applied.
The Supplementary file reports the probe identifier (cg number) used in the custom Infinium array (HorvathMammalMethylChip40). The weights used in this linear combination are specified in the respective column entitled "Coef.".
The formula assumes that the DNA methylation data measure "beta" values, but the formula could be adapted to other ways of generating DNA methylation data.

General description of age transformation

The human-rat clocks for chronological age used log linear transformations that are similar to those employed for the HUMAN pan tissue (Horvath 2013, Genome Biology).

An elastic net regression model (implemented in the glmnet R function) was used to regress a transformed version of age on the beta values in the training data. The glmnet function requires the user to specify two parameters (alpha and beta). Since we used an elastic net predictor, alpha was set to 0.5. But the lambda value of was chosen by applying a 10 fold cross validation to the training data (via the R function cv.glmnet).

The elastic net regression results in a linear regression model whose coefficients b_0, b_1, . . . , relate to transformed age as follows
$F(\text{chronological age})=b_0+b_1 CpG_1+ . . . +b_p CpG_p+\text{error}$

Note that the intercept term is denoted by b_0. The coefficient values can be found in the Supplementary file. Based on the coefficient values from the regression model, DNAmAge is estimated as follows

$$DNAm\text{Age}=F^{-1}(b_0+b_1 CpG_1+ . . . +b_p CpG_p)$$

, where $F^{-1}(y)$ denotes the mathematical inverse of the function F(.). Thus, the regression model can be used to predict to transformed age value by simply plugging the beta values of the selected CpGs into the formula.

Defining Properties of the log linear transformation

As indicated by its name, the “log-linear” function, has a logarithmic dependence on age before the average age of sexual maturity (of the species) and a linear dependence after Age at Sexual Maturity (of the species). For the human-rat clocks we used the following averages at sexual maturity (in units of years): 13.5 years for humans and 0.219178082 years for rats (**Supplementary Table**).

bioRxiv preprint doi: https://doi.org/10.1101/2023.08.06.552148; this version posted August 7, 2023. The copyright holder for this preprint (which was not certified by peer review) is the author/funder, who has granted bioRxiv a license to display the preprint in perpetuity. It is made available under aCC-BY 4.0 International license.

We used a piecewise transformation, parameterized by Age of Sexual Maturity (A). The transformation is F(x), given by

$$F(x) = g\left(\frac{x+1.5}{A+1.5}\right) \text{ where } g(t) = \begin{cases} \log(t), & for\ 0 \le t \le 1 \\ t-1, & for\ 1 \le t \end{cases}$$

Explicitly, F(x) is given by

$$F(x) = \begin{cases} \log\left(\frac{x+1.5}{A+1.5}\right), & for\ 0 \le x \le A \\ \frac{x-A}{A+1.5}, & for\ A \le x \end{cases}$$

In order to use this transformation to predict Age on <u>*new samples*</u>, one needs to use the *inverse* transformation, $F^{-1}(y)$, given by

$$F^{-1}(y) = \begin{cases} (A+1.5) * \exp(y) - 1.5, & for\ y \le 0 \\ (A+1.5)y + A, & for\ y \ge 0 \end{cases}$$

For predicting age, apply the inverse transformation to coefficient-weighted sum. That is,

$$DNAmAge = F^{-1}(x * \beta)$$

where β is the vector of coefficients and x is the vector of methylation values, with an intercept term.

R Implementation of the log linear transformation

```
### Applies the log linear transformation to the input vector x, i.e. to
Age
F= Vectorize(function(x, maturity, ...) {
  if (is.na(x) | is.na(maturity)) {return(NA)}
  k <- 1.5
  y <- 0
  if (x < maturity) {y = log((x+k)/(maturity+k))}
  else {y = (x-maturity)/(maturity+k)}
  return(y)
})
### Inverse log linear transformation
F.inverse= Vectorize(function(y, maturity, ...) {
  if (is.na(y) | is.na(maturity)) {return(NA)}

  k <- 1.5
  x <- 0
  if (y < 0) {x = (maturity+k)*exp(y)-k}
  else {x = (maturity+k)*y+maturity}
  return(x)
})
```

The DNAm Age estimate is estimated in two steps.

First, one forms a weighted linear combination of the CpGs whose details can be found in Table

The table reports the probe identifier (cg number) used in the custom Infinium array (HorvathMammalMethylChip40). The weights used in this linear combination are specified in the respective column entitled "Coef.".

The formula assumes that the DNA methylation data measure "beta" values but the formula could be adapted to other ways of generating DNA methylation data.

Pseudo R code

```
# R function for multivariate regression model
multivariatePredictorCoef=function(dat0, datCOEF,imputeValues=FALSE)
{
```

bioRxiv preprint doi: https://doi.org/10.1101/2023.08.06.552148; this version posted August 7, 2023. The copyright holder for this preprint (which was not certified by peer review) is the author/funder, who has granted bioRxiv a license to display the preprint in perpetuity. It is made available under aCC-BY 4.0 International license.

```
datout=data.frame(matrix(NA,nrow=dim(dat0)[[2]]-
1,ncol=dim(datCOEF)[[2]]-1 ))
match1=match(datCOEF[-1,1],dat0[,1] )
if (   sum(!is.na(match1))==0 ) stop("Input error. The first column
of dat0 does not contain CpG identifiers (cg numbers).")
dat1=dat0[match1,]
row.names1=as.character(dat1[,1])
dat1=dat1[,-1]
if (imputeValues ){dat1=impute.knn(data=as.matrix(dat1) ,k =
10)[[1]]}
for (i in 1:dim(dat1)[[2]] ){ for (j in
2:dim(as.matrix(datCOEF))[[2]] ){
datout[i,j-1]=sum(dat1[,i]* datCOEF[-1,j],na.rm=TRUE)+
datCOEF[1,j]}}
colnames(datout)=colnames(datCOEF)[-1]
rownames(datout)=colnames(dat0)[-1]
datout=data.frame(SampleID= colnames(dat0)[-1],datout)
datout
} # end of function

# read in supplementary table
datCoef=read.csv("TableS.csv")

The first columns should read as follows
names(datCoef)
   1) var
   2) Coef.PrelimRatPanTissue
   3) Coef.PrelimRatBloodTissue
   4) Coef.PrelimRatBrainTissue
   5) Coef.PrelimRatLiverTissue
   6) Coef.PrelimHumanRatPanTissueLogLinearAge
   7) Coef.PrelimHumanRatBloodTissueLogLinearAge
   8) Coef.PrelimHumanRatRelativeAgePan
   9) Coef.PrelimHumanRatRelativeAgeBlood
   10)      Coef.FinalRatPanTissue
   11)      Coef.FinalRatBloodTissue
   12)      Coef.FinalRatBrainTissue
   13)      Coef.FinalRatLiverTissue
   14)      Coef.FinalHumanRatPanTissueLogLinearAge
   15)      Coef.FinalHumanRatBloodTissueLogLinearAge
   16)      Coef.FinalHumanRatRelativeAgePan
   17)      Coef.FinalHumanRatRelativeAgeBlood
ETC ETC


# Restrict attention to the FINAL rat clocks columns
datCoef=datCoef[,c(1,10:17)]

# assume the first column of dat0 contains the CpG identifiers
match1=match(datCoef[-1,1],dat0[,1] )
missingProbes= as.character(datCoef[-1,1] )[is.na(match1)]

dat1=dat0[match1,]
# data frame with predicted values.
datPredictions=multivariatePredictorCoef(dat1,datCOEF=datCoef,impute
Values=FALSE)
```

bioRxiv preprint doi: https://doi.org/10.1101/2023.08.06.552148; this version posted August 7, 2023. The copyright holder for this preprint (which was not certified by peer review) is the author/funder, who has granted bioRxiv a license to display the preprint in perpetuity. It is made available under aCC-BY 4.0 International license.

```
#let's relabel the columns by replacing "Coef" with "DNAm" since the
columns contain estimates of age or relative age instead of
coefficient values

colnames(datPredictions)=gsub(pattern="Coef", replacement="DNAm",
x=colnames(datPredictions))

# We need to transform the human rat clock for chronological age
using the inverse of the log linear #transformation.
For rats, the age at sexual maturity has to be set to 0.21917808
years.
datPredictions$DNAm.FinalHumanRatPanTissueLogLinearAge=
F.inverse(datPredictions$DNAm.FinalHumanRatPanTissueLogLinearAge,
maturity=0.21917808)
datPredictions$DNAm.FinalHumanRatBloodTissueLogLinearAge=
F.inverse(datPredictions$DNAm.FinalHumanRatBloodTissueLogLinearAge,
maturity=0.21917808)


#The data frame "datPredictions" contains the age estimates in units
of years and relative age estimates.
```

1 Arneson, A. *et al.* A mammalian methylation array for profiling methylation levels at conserved sequences. *Nature Communications* **13**, 783 (2022). https://doi.org:10.1038/s41467-022-28355-z
